# I-a^low^CD11b^high^ DC Regulates the Immune Response in the Eyes of Experimental Autoimmune Uveitis

**DOI:** 10.1155/2020/6947482

**Published:** 2020-03-10

**Authors:** Yu Zhao, Jingwen Wang, Zhang Min, Li Peng, Shuhong Qi, Wei Lin

**Affiliations:** ^1^Institute of Basic Medicine, Shandong Provincial Hospital Affiliated to Shandong First Medical University, Institute of Basic Medicine, Shandong First Medical University & Shandong Academy of Medical Schools, Jinan City 250062, China; ^2^The Third Affiliated Hospital of Shandong First Medical University, Affiliated Hospital of Shandong Academy of Medical Schools, 38# Wuyingshan Road, Jinan City 250031, China; ^3^Departments of Medicine, Tibet Nationalities University, Xian City 712082, China; ^4^Britton Chance Center for Biomedical Photonics, Wuhan National Laboratory for Optoelectronics-Huazhong University of Science and Technology, Wuhan, Hubei 430074, China; ^5^MoE Key Laboratory for Biomedical Photonics, Collaborative Innovation Center for Biomedical Engineering, School of Engineering Sciences, Huazhong University of Science and Technology, Wuhan, Hubei 430074, China

## Abstract

Regulatory dendritic cells (DC_reg_) have been reported to be a negative regulator in the immune response. These cells are widely distributed in the liver, spleen, and lung. However, the status and function of DC_reg_ in the eyes and disease are still not very clear. Herein, we found that the number of I-a^low^CD11b^high^ DC increased in the eye and spleen at the recovery stage of experimental autoimmune uveitis (EAU), which is a mouse model for autoimmune uveitis. These cells expressed lower levels of CD80, CD86, and CD54 than the mature DCs and expressed interleukin 10 (IL-10), indoleamine 2,3-dioxygenase (IDO), and transforming growth factor beta (TGF-*β*) as well. Moreover, these DC_reg_ can regulate the development of EAU by promoting CD4^+^CD25^+^Foxp3^+^ regulatory T cells. The increased interferon-gamma (IFN-*γ*) in the aqueous humor of EAU participates in inducing DC_reg_ to alleviate the symptom of EAU. Furthermore, DC_reg_ was found to exist in the eyes of normal mice. Aqueous humor, containing a certain concentration of IL-10, TGF-*β*, prostaglandin E2 (PGE2), IDO, and nitric oxide (NO), induced the tolerance of DC_reg_ in normal eyes. It can be concluded that DC_reg_ exists in the eyes and plays a protective role in inflamed eyes. These DC_reg_ induced by IFN-*γ* might be used as a strategy to develop therapy for EAU management.

## 1. Introduction

Dendritic cells (DCs) have been identified as very effective antigen presenting cells (APCs) with the apparently unique ability to prime and to activate naive T lymphocytes [1, 2]. Mature DCs typically express high levels of “activation” markers (major histocompatibility complex II (MHC-II), CD54, CD80, and CD86) and possess potent T-cell activation ability [3]. In addition, immature DCs express low levels of “activation” markers and have high endocytic capacity, whereas regulatory DCs with regulatory functions have been defined to control T-cell responses [3, 4]. I-a^low^CD11b^high^ DCs have been characterized as a subset of regulatory DCs. They can suppress T-cell proliferation by inducing nitric oxide (NO) [5] or by inducing CTLA-4-dependent (cytotoxic lymphocyte antigen 4-dependent) interleukin 10 (IL-10) secretion and indoleamine 2,3-dioxygenase (IDO) expression in tumors [6]. Different subsets of DCs may play different roles during different developmental/functional stages [7]. Regulatory DCs can balance the immune response and are present in several organs (e.g., lung, spleen, and liver) [5, 8–10]. Recently, DCs were also found to exist in the eyes [11–13], which is considered to be an immune-privileged tissue. But the role and the subsets of DCs in the eyes are still unclear.

To date, regulatory DCs (DC_reg_) were generated by culturing DCs in the presence of immunosuppressive cytokines, such as IL-10 and transforming growth factor beta (TGF-*β*) or in the presence of immunomodulatory drugs [4, 5, 8, 14, 15]. Several studies have shown that the microenvironment in certain tissues has an ability to induce DC development and also affects the function of DC [5, 8–10, 16]. Previous studies demonstrated that splenic microenvironment or lung microenvironment could drive mature DCs or stem cells to differentiate into DC_reg_ [5, 16–18]. Several factors participate in the regulation of DCs status, such as IL-10, TGF-*β*, interferon gamma (IFN-*γ*), and/or other compounds such as vitamin D receptor ligands, vasoactive intestinal peptide, and thymic stromal lymphopoietin (TSLP) [4, 5, 8, 14, 15, 19]. In the eyes, aqueous humors are produced by ciliary epithelial cells and contain NO, IDO, prostaglandin E2 (PGE2), and TGF-*β* [20–22], which play a significant role in promoting anti-inflammatory and tolerogenic activity. Thus, aqueous humors may influence the status of DCs in the eyes, but there are no experiments to confirm this.

Uveitis is an ocular disease, which can cause blindness in humans [23, 24]. This disease correlates with immune disorders, including increasing CD4^+^ T cells infiltration in the eyes [25–28]. Uveitogenic antigen-specific CD4^+^ T cells have been believed to be crucial effectors to infiltrate in the sites of inflammatory eyes to drive inflammation and tissue damage [25, 27, 29]. DCs act as a unique antigen presenting cells and activate naïve T cells, which are also involved in the pathogenic process of uveitis [11, 12, 30, 31]. DCs exist in the peripheral margins and juxtapapillary areas of the retina [12]. Functional mature DCs have been found in the choroid [30] and are believed to cause antigen-specific Th1 or Th17 cells to induce the development of experimental autoimmune uveoretinitis (EAU) [11]. Impairing the maturation of DCs with the drug could prevent the generation of antigen-specific Th1 or Th17 cells to attenuate EAU [32]. Regulatory bone marrow-derived dendritic cells, which induced in vitro, suppressed the development of EAU [33]. However, the status of DCs in uveitis and the regulatory roles of DCs are still not very clear.

The EAU mouse model is a well-established rodent model used for human autoimmune uveitis induction and contains specific self-renewal characteristics [34]. Based on this model, we investigated the phenotype and subsets of DCs in the eyes and analyzed the roles of regulatory DCs in the development of EAU. Furthermore, we explored the mechanism affecting the differentiation of regulatory DCs in the eyes.

## 2. Materials and Methods

### 2.1. Animal Experiment

Pathogen-free female C57BL/6J (6- to 8-weeks-old) mice were purchased from Beijing Vital River Laboratory Animal Technology Co., Ltd. (Beijing, China). C57Lan/J (B6 CD11c-DTR-GFP) mice and CD45.1-expressing mice were purchased from the Jackson Laboratory (Bar Harbor, ME, USA). These mice were maintained in specific pathogen-free conditions, and all experimental procedures were licensed by our local regulatory agency (Shandong Academy of Medical Sciences, Jinan, China, SYXK 20180007). Mice were allocated randomly to cages with *n* = 4-6 mice per group according to the individual experimental group. EAU in C57BL/6 mice was inducted by the 350 *μ*g of human interphotoreceptor retinoid-binding protein peptide (IRBP)_1-20_ (China Peptides Co., Ltd., Shanghai, China) emulsified in complete Freund's adjuvant with mycobacterium (CFA, Sigma-Aldrich, St. Louis, MO, USA). A total of 500 ng of Pertussis toxin (PTX, Enzo Life Sciences, Farmingdale, YN, USA) was intraperitoneally injected at the footpad, neck, two sides, and tail at six points for every mouse as previously described [35]. After immunization, the mice were examined every four days by Genesis-D camera (Kowa Company Ltd., Japan) for the evaluation of the clinical scores [35]. The eyes were obtained after sacrificing every four days, and the hematoxylin and eosin (H&E) staining was performed for the assessment of the pathological scores [35–38].

### 2.2. Depletion of DCs

Diphtheria toxin (DT, 5 ng/eye) was used to delete the CD11c cells in the eyes of CD11c-DTR-GFP mice by subconjunctival injection. This approach resulted in 95% depletion of CD11c cells and lasted for 72 h. After DT treatment for 24 h, the antigens were administered to induce EAU. The symptoms and severity of the inflammation in CD11c-DTR mice were evaluated by the histopathological scores every four days [11, 39].

### 2.3. Isolation of Cells

For ocular cell collection, the eyes were collected from the mice as reported previously [40]. Briefly, the eyes were obtained from naive and EAU mice. Following the removal of the lens and the cornea from the eyes, a single-cell suspension of the eyes was prepared by digestion for 10 min at 37°C with collagenase (1 mg/ml) and deoxyribonuclease (DNAse, 100 *μ*g/ml) in RPMI-1640. The eye-infiltrating cells were subsequently obtained.

Spleen cells were obtained from naive and EAU mice following immunization. Red blood cell lysis buffer (Beijing Solarbio Science & Technology Co., Ltd., Beijing, China) was used to lyse the red blood cells, and the cells were collected by Ficoll-Hypaque density gradient centrifugation. The suspension was cultured at 37°C in a 5% CO_2_ incubator for flow cytometry analysis.

For isolation of primary CD4^+^ T cells, a CD4 negative-selection kit (Miltenyi Biotec, Bergisch Gladbach, Germany) was separately used, and the cells were cultured as previously reported [41, 42]. I-a^low^CD11b^high^ DCs (DC_reg_) or CD11c^+^I-a^high^ DCs (DC_m_), which were excluded from macrophages and monocytes, were isolated by a cell sorting instrument (BD FACSAria™ III, BD Biosciences, CA, USA).

In the presence and/or absence of the aqueous humor stimulation, the isolated ocular dendritic cells were cultured in media, supplemented with rGM-CSF (20 ng/ml), rIL-4 (5 ng/ml), and rFLT3L (200 ng/ml, all from R&D Systems, Minneapolis, MN,USA) [43, 44] for 3 days. Using Horizon fixable viability dye staining, the viable cells were detected by flow cytometry.

### 2.4. Adoptive Transfer Experiments

I-a^low^CD11b^high^ DC_reg_ or I-a^high^CD11b^low^ DC_m_, which were excluded from macrophages and monocytes, were isolated as previously described [5, 45]. The isolated cells (5 × 10^5^) were administered to an EAU mouse, which were immunized for 8 days by an intravenous injection. After transferring for 8 days, the mice were euthanized, and the eyes and spleens were harvested for H&E staining and flow cytometry analysis.

### 2.5. IFN-*γ* Treatment or Neutralizing Anti-IFN-*γ* Antibody Treatment

For IFN-*γ* treatment, DCs were isolated from EAU mice and were pretreated with IFN-*γ* (100 U/ml) for 48-72 h. These DCs were washed twice with phosphate buffer saline (PBS) and were analyzed using FACSuite or collected for animal transfer (5 × 10^5^/mouse).

To assess the effects of IFN-*γ* on DCs, 2 *μ*g/ml neutralizing anti-IFN-*γ* antibodies were added in wild-type DC culture medium or in the DC culture medium with aqueous humor stimulation to neutralize autosecreting IFN-*γ*. The mice were injected intraperitoneally with anti-IFN-*γ* neutralization antibody (Abcam company, Cambridge, MA, USA) or control antibody mouse IgG (250 *μ*g per mouse) every other day following immunization for a total period of 8 days.

### 2.6. Antibodies and Flow Cytometry

Fluorescent antibodies of (PE-cy5)-conjugated CD3*ε* (clone 145-2C11), (FITC)-conjugated CD4 (clone GK1.5), (PE)-conjugated CD25 (clone PC61.5), (BV711)-conjugated CD11b (clone M1/70), (APC)-conjugated CD80 (clone 16-10A), (APC)-conjugated CD86 (clone GL1), (APC)-conjugated CD54 (clone 3E2), (PE)-conjugated I-a (clone M5/114.15.2), (APC-cy7)-conjugated CD11c (clone N418), (APC)-conjugated dendritic cell marker DCIR2 monoclonal antibody (33D1), (percp)-conjugated CD45 (clone 30-F-11), (Alexa Fluor 700)-conjugated CD45.1 (clone A20), (BV421)-conjugated CD64 (clone X54-5/7.1), (BV650)-conjugated F4/80 (clone BM8), (PE-cy5)-conjugated CD19 (clone eBio 1D3), (PE-cy5)-conjugated NK1.1 (clone PK136), (FITC)-conjugated CD26 (clone H194-112), (PE-cy7)-conjugated CD69 (clone H1.2F3), (APC)-conjugated Ki67 (clone 7B11), (percp-cy5)-conjugated TGF-*β* (clone TW7-20B9), (APC)-conjugated IL-10 (clone JES5-16E3), (APC)-conjugated Foxp3 (clone FJK-16S), (APC)-conjugated IL-17 (clone eBio64DEC17), and (APC)-conjugated IFN-*γ* (clone XMG1.2) conjugated with the corresponding fluorescent dyes were purchased from eBioscience (San Diego, CA, USA) and BioLenged (San Diego, CA, USA). Single-cell suspensions (1 × 10^6^ cells) were stained with different monoclonal antibodies, according to the protocol provided by the manufacturer for the corresponding antibodies. Subsequently, each sample was analyzed using FACSuite and the CellQuest data acquisition and analysis software (BD Biosciences, CA, USA). To assess intracellular cytokine expression, the prepared cells were prestimulated with leukocyte activation cocktail, with BD GolgiPlug™ (BD Biosciences, CA, USA) for 5 h, at 37°C in a 5% CO_2_ environment, and were subsequently incubated with fluorescent labeled antibody, according to the manufacturer's instructions. Foxp3 was stained according to the protocol of Foxp3 staining Kit (eBioscience Inc., San Diego, CA, USA). BD Horizon fixable viability stain (eBioscience Inc., San Diego, CA, USA) was used to rule out nonviable cells.

### 2.7. Cytokine Levels in the Serum, Aqueous Humor, and the Supernatant of Cells

Blood samples were collected and incubated at room temperature and centrifuged at 1,000 × g. The aqueous humors were obtained from the eyes of EAU mice or wild type mice by fine needle aspiration. Ten mice for each group were used, and the obtained aqueous humor was mixed together for testing. The level of IFN-*γ*, IL-10, and TGF-*β* in aqueous humors was quantified by using ELISA kits (Elabscience Biotechnology Co., Ltd., Wuhan, China). NO production was measured as the nitrite concentration using the Griess assay [5]. PGE2 (R&D Systems Inc., MN, USA) and IDO were detected by an ELISA kit (Invitrogen, MA, USA).

### 2.8. DC-T Cell Coculture

CD4^+^ T cells were purified from the spleen of IRBP_1-20_-immunized B6 mice following 16 days and were stimulated with IRBP_1-20_ (10 *μ*g/ml) in the presence of 1 × 10^6^ irradiated syngeneic spleen cells as APCs for 72 h, and then antigen-specific T cells were obtained by magnetic beads (CD4^+^ T cells isolation kits, Miltenyi Biotec, Bergisch Gladbach, Germany). Subsequently, CD4^+^ T cells were cocultured with antigen-pulsed mature DCs (T cell : DC_m_ = 10 : 1) for 48 h. DC_reg_ were isolated by cell sorting methodologies and were added to the coculturation DC-T (T : DC_reg_ : DC_m_ = 10 : 1 : 1). The status of T cells was analyzed by flow cytometry.

Neutralization Abs to mouse IL-10 (5 *μ*g/ml, Clone # JES052A5) and TGF-*β* (5 *μ*g/ml, Clone # 1D11R) were purchased from R&D Systems (MN, USA). IDO inhibitor 1-methyltryptophan (1 mM), PGE2 inhibitor indomehacin (40 *μ*M), and inducible NO synthase (iNOS) inhibitor 1,4 PBIT (S,S′-1,4-phenylene-bis (1,2-ethanediyl) bis-isothiourea, dihydrobromide, 5 *μ*g/ml) were added to the experimental cultures, respectively.

### 2.9. Statistical Analysis

The data were analyzed using GraphPad Prism 5 software (GraphPad, San Diego, CA). Each experiment was carried out in duplicate and was repeated three times. The clinical and histological EAU data are usually statistically analyzed using Mann-Whitney *U*-test for two groups or Kruskal-Wallis Test for more than two groups. Two-tailed Student's *t*-test or one-way analysis of variance (ANOVA) was applied for the normal distribution of datasets. The data were represented as mean ± standard error of the mean (SEM). *P* values < 0.05 (^∗^), 0.01 (^∗∗^), and 0.001 (^∗∗∗^) were considered for significant differences.

## 3. Results

### 3.1. The Number of I-a^low^CD11b^high^ DCs Increased in the Recovery Stage of the EAU Model

**Figure 1:**
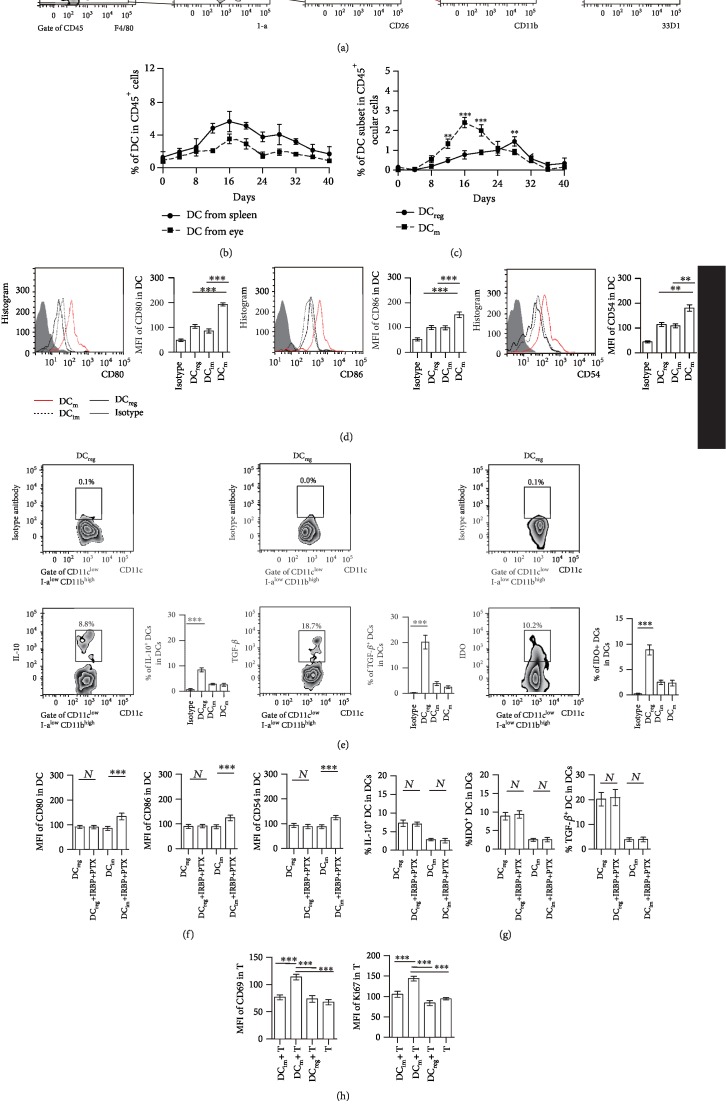
The states of I-a^low^CD11b^high^ DCs in the eyes of EAU. (a) The representative diagram of I-a^low^CD11b^high^ DCs (DC_reg_) or I-a^high^CD11b^low^ DCs (DC_m_) in the eyes of EAU after being immunized for 28 days. (b) Dynamic change of percentage of DC in CD45^+^ cells in the eyes of EAU. (c) Dynamic change of percentage of DC_reg_ and DC_m_ in CD45^+^ cells in the eyes of EAU. Three mice were used for every group; the experiment was replicated three times; data were presented as mean ± standard error of the mean (SEM), Kruskal-Wallis test, and ^∗∗∗^*P* < 0.001 and ^∗∗^*P* < 0.01. (d) The expression of CD80, CD86, and CD54 on I-a^low^CD11b^high^ DCs (DC_reg_), I-a^low^CD11b^low^ DCs (DC_im_), and I-a^high^CD11b^low^ DCs (DC_m_). MFI is the mean intensity of fluorescence of molecules on the surface of DCs. (e) The representative intracellular cytokines in DC_reg_ (upper panels) and the expression of intracellular cytokines in DC_reg_ compared with those in DC_im_ and DC_m_. (d, e) DC_reg_, DC_im_, and DC_m_ were isolated from the eyes of EAU, and experiments were replicated three times; data were presented as mean ± standard error of the mean (SEM) and ^∗^*P* < 0.05, ^∗∗^*P* < 0.01, and ^∗∗∗^*P* < 0.001. (f) The surface expression of CD80, CD86, and CD54 in DC_reg_ and DC_im_, which were stimulated by 10 ng/ml of IRBP_1-20_ and 10 ng/ml of PTX (to induce maturation) for 24-48 h, was measured by flow cytometry. The mean fluorescence intensity (MFI) is shown as mean ± standard error of the mean (SEM) from three separate experiments. (g) The percentage of IL-10^+^ DCs, IDO^+^ DCs, or TGF-*β*^+^ DCs in DC_reg_ or DC_im_, which were stimulated by IRBP and PTX. (h) Isolated DC_m_ promoted CD4^+^ T cells to express a higher level of CD69 and Ki67 than DC_reg_ or DC_im_ did. (f–h) were analyzed with one-way ANOVA test. ^∗∗∗^*P* < 0.001; *N* is not significantly different.

To study the status of DCs in the eyes of EAU, the EAU mice model was established, and the status of DCs in the inflamed eyes was detected. The phenotype of ocular DCs was detected according to a previous report [45]. The macrophages were discriminated from DCs by CD64 and F4/80 expression. In addition to CD64^+^F4/80^+^ macrophages and monocyte-derived cells, CD3^+^ T cells, CD19^+^B220^+^ B cells, and NK1.1^+^ natural killer (NK) cells were next excluded from the analysis using a “lineage mix,” and the remaining cells were gated based on the expression of I-a molecules (Lineage^−^I-a^+^ cells). CD11c^+^CD26^+^DCs were further used to analyze the percentage of DCs in eyes (Figure 1(a)). CD11b and I-a were used to identify the subsets of mature DCs (CD11c^+^CD26^+^I-a^high^CD11b^low^ DCs, DC_m_), immature DCs (CD11c^+^CD26^+^I-a^low^CD11b^low^ DCs, DC_im_), and regulatory DCs (CD11c^+^CD26^+^I-a^low^CD11b^high^ DCs, DC_reg_) (Figure 1(a)). DC_reg_ expressed 33D1, a marker of ocular DCs [12]. The number of ocular DCs within the inflamed eyes increased on the 8th day, and the first peak appeared on the 16th day (initiation stage of EAU, Figure 1(b)), which occurred earlier than the majority of the serious pathological changes in the eye. These changes appeared from the 16th-24th day following immunization. Subsequently, the second peak of DCs occurred on the 28th day (recovery stage, Figure 1(b)). Next, the subsets of ocular DCs during the process of EAU were analyzed. From the 12th to the 20th day, the majority of increased DCs were DC_m_. The increased number of DC_m_ were higher than that of the DC_reg_ (Figure 1(c)). On the 28th day, the number of the increased ocular DC_reg_ was higher than that of DC_m_ (Figure 1(c)). Similar results were found in the splenic cells of the EAU mice (Supplementary [Supplementary-material supplementary-material-1]). These results indicated that DC_reg_ might participate in the recovery stage of EAU.

DC_reg_ were obtained from the eyes of the animals on the 28th day postimmunization, and these cells expressed lower levels of CD80, CD86, and CD54, and higher levels of IL-10, IDO, and TGF-*β* compared with those noted in DC_m_ (Figures 1(d) and 1(e)). Furthermore, with 10 ng/ml of IRBP and 10 ng/ml of PTX stimulation, the isolated ocular DC_reg_ could not became mature to express high levels of CD80, CD86, and CD54 (Figure 1(f)). The expression levels of IL-10, IDO, and TGF-*β* in DC_reg_ did not significantly change in DC_reg_ following IRBP and PTX stimulation compared with DC_reg_ in the absence of antigen stimulation (Figure 1(g)). Moreover, these DC_reg_ could not promote CD4^+^ T cells to express high level CD69 and Ki67 but DC_m_ could (Figure 1(h)). These data indicated that DC_reg_ could not be activated, and those cells might play a regulatory role in the eyes.

### 3.2. I-a^low^CD11b^high^ DCs Promote CD4^+^CD25^+^Foxp3^+^ T Cells to Alleviate the Symptom of EAU

**Figure 2:**
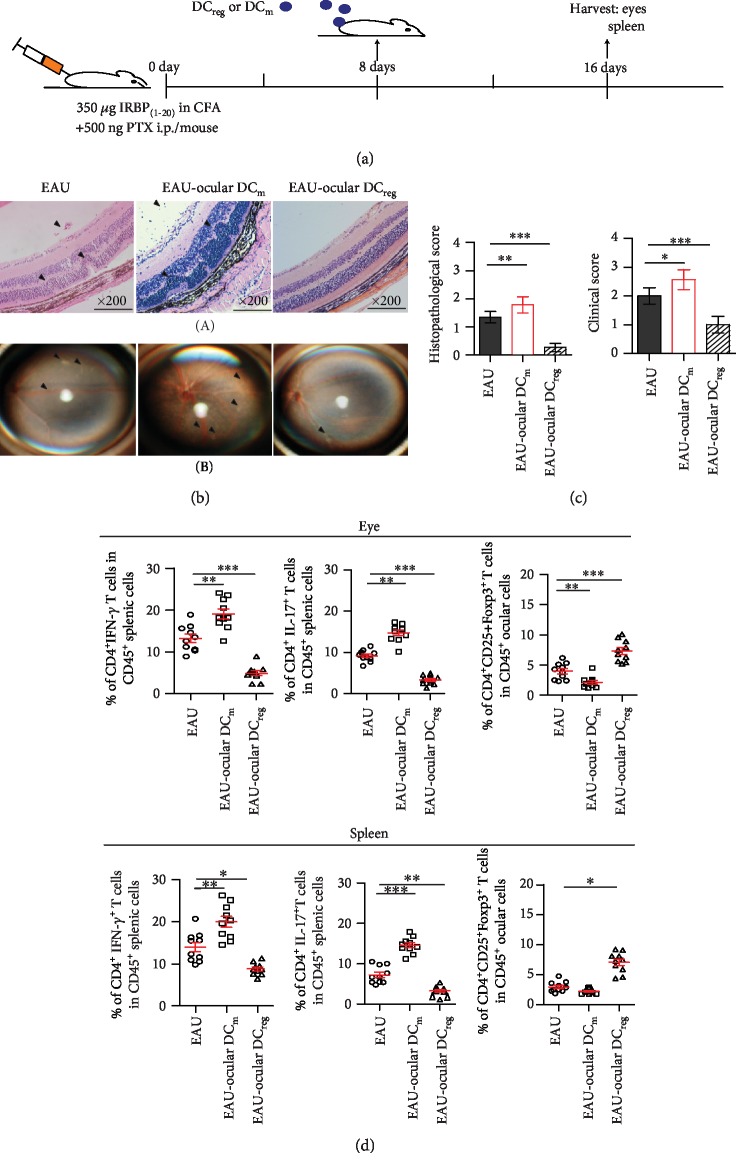
The role of ocular DC_reg_ in the EAU. (a) Diagram of DC_reg_ or DC_m_ transferred into immunized mice is as shown. (b) Histopathological damage of eyes (A) and clinical symptom (B) was assessed in DC_reg_-transferred mice compared with that of nontreated EAU mice or DC_m_-transferred mice by H&E staining and funduscopy on the 16th d of postimmunization. H&E staining of the retina at 200x magnification. Black arrows mark infiltrating lymphocytes and retinal disorganization (A). Scale bar = 100 *μ*m. Multifocal chorioretinal lesions, severe vacuities, and linear lesions were observed on the eyes of EAU mice and DC_m_-transferred mice (B, black arrows). (c) The histopathological and clinical scores were evaluated in DC_reg_- or DC_m_-transferred mice compared with that of nontreated EAU mice (the experiments were replicated three times, and a total of 10 mice/group were used. Data were presented as mean ± standard error of the mean (SEM), ANOVA test, and ^∗^*P* < 0.05, ^∗∗^*P* < 0.01, and ^∗∗∗^*P* < 0.001). (d) The percentage of CD4^+^IFN-*γ*^+^ T cells, CD4^+^IL-17^+^ T cells, or CD4^+^CD25^+^Foxp3^+^ T cells in CD45^+^ lymphocytes of eyes and spleen of ocular DC_reg_- or DC_m_-transferred mice compared with that of nontreated EAU mice (the experiments were replicated three times, and a total of 10 mice/group were used. Data were presented as mean ± standard error of the mean (SEM), ANOVA test, and ^∗^*P* < 0.05, ^∗∗^*P* < 0.01, and ^∗∗∗^*P* < 0.001).

To analyze the role of ocular DC_reg_ in the development of EAU, DC_reg_ or DC_m_ were isolated from the inflamed eyes of the EAU on the 28th day postimmunization with a cells sorting instrument and were transferred into EAU mice (Figure 2(a), 5 × 10^5^/mice, for three mice/every group were transferred). The severity of EAU was analyzed on the 8th day following DC_reg_ transfer. The retinal damage was slighter in the eyes of DC_reg_-transferred mice than other group (Figure 2(b)). Both clinical and histopathological scores of the eyes of DC_reg_-transferred mice decreased compared with those noted in the EAU mice (Figure 2(c)). However, DC_m_ transfer aggravated the symptom of EAU (Figures 2(b) and 2(c)).

The number of CD4^+^IFN-*γ*^+^ T and CD4^+^IL-17^+^ T cells derived from the eyes or spleen of DC_reg_-transferred mice decreased compared with the cells derived from the EAU without transferring (Figure 2(d)). However, the number of CD4^+^CD25^+^Foxp3^+^ T cells from the eyes of DC_reg_-transferred mice was higher than these cells from EAU without transferring. Similar results were found in the spleen of DC_reg_-transferred mice (Figure 2(d)). However, in DC_m_-transferred mice, the number of CD4^+^IFN-*γ*^+^ T cells and CD4^+^IL-17^+^ T cells from the eyes or spleen was higher than that derived from EAU animals without DC_m_ transfer, but the number of CD4^+^CD25^+^Foxp3^+^ T cells derived from the spleen of DC_m_-transferred mice was not significantly different from that without transferred (Figure 2(d)). All the above data indicated that increased DC_reg_ in the inflamed eyes can alleviate the symptom of EAU by inducing CD4^+^CD25^+^Foxp3^+^ T cells.

### 3.3. I-a^low^CD11b^high^ DCs Promote the Differentiation of CD4^+^CD25^+^Foxp3^+^ T Cells In Vitro

**Figure 3:**
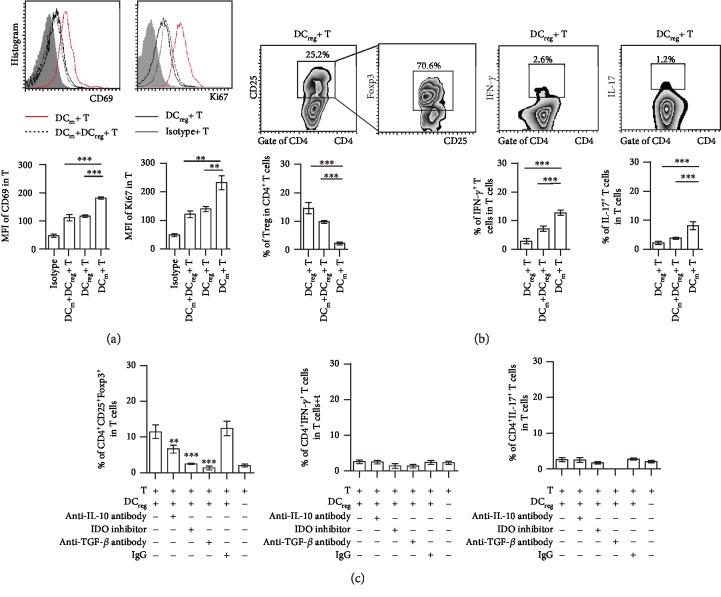
DC_reg_ influence the activation of T cells and induce CD4^+^CD25^+^Foxp3^+^ T cells *in vitro*. (a) The expression of CD69 and Ki67 on the T cells cocultured with DC_reg_, DC_m_, and both of them. DC_reg_ and DC_m_ were isolated from the eyes of EAU and cocultured with T cells from EAU (DC : T = 1 : 10) for 24-48 h. (b) The percentage of CD4^+^IFN-*γ*^+^ T cells, CD4^+^IL-17^+^ T cells, or CD4^+^CD25^+^Foxp3^+^ T cells in T cells cocultured with DC_reg_, DC_m_, and both of them. (c) With blocking IL-10, IDO, and TGF-*β* alone in the coculturation of T cells and DC_reg_ or not, the percentage of CD4^+^CD25^+^Foxp3^+^ T cells, CD4^+^IFN-*γ*^+^ T cells, or CD4^+^IL-17^+^ T cells compared with those without treatment (experiments were replicated three times, data were presented as mean ± standard error of the mean (SEM), ANOVA test, and ^∗^*P* < 0.05, ^∗∗^*P* < 0.01, and ^∗∗∗^*P* < 0.001).

To further analyze the mechanisms of DC_reg_ in regulating the symptoms of EAU, DC_reg_ and DC_m_ were isolated from the eyes of EAU animals and cocultured with isolated T cells, separately. DCs were pulsed with 10 ng/ml of IRBP_1-20_ and 10 ng/ml of PTX for 24 h, and subsequently, CD4^+^ T cells were isolated from EAU animals and cocultured with these DCs for 48 h. The activation and proliferation of T cells were analyzed by flow cytometry with the markers CD69 and Ki67, separately. T cells, which were cocultured with DC_m_, expressed high levels of CD69 and Ki67 than T cells cocultured with DC_reg_ (Figure 3(a)). Moreover, DC_reg_ decreased the expression of CD69 and Ki67 in T cells, which were cocultured with DC_m_ and DC_reg_ (Figure 3(a)). T cells cocultured with DC_reg_ expressed higher levels of CD25 and Foxp3 than T cells cocultured with DC_m_ (Figure 3(b)). The added of DC_reg_ could increase the percentage of CD4^+^CD25^+^Foxp3^+^ T cells in the coculture of DC_m_ and T cells (Figure 3(b)). However, DC_m_ cocultured with T cells promoted the induction of higher expression levels of CD4^+^IFN-*γ*^+^ T cells and CD4^+^IL-17^+^ T cells than these cocultured with DC_reg_ (Figure 3(b)). Adding DC_reg_ could decrease the percentage of CD4^+^IFN-*γ*^+^ T cells or CD4^+^IL-17^+^ T cells in the coculture of DC_m_ and T cells (Figure 3(b)).

Blocking of IDO, IL-10, and TGF-*β* with the corresponding neutralizing antibody or inhibitor, the percentage of CD4^+^CD25^+^Foxp3^+^ T cells in the coculture of DC_reg_ and T cells was decreased (Figure 3(c)). However, the use of neutralizing antibodies and inhibitors could not affect the expression levels of IFN-*γ* or IL-17 in CD4^+^ T cells (Figure 3(c)). The secretion of the soluble factors by DC_reg_ might be correlated with the induction of CD4^+^CD25^+^Foxp3^+^ T cells.

### 3.4. I-a^low^CD11b^high^ DC_reg_ Derived from the Inflamed Spleen Inhibit the Inflammation of EAU

**Figure 4:**
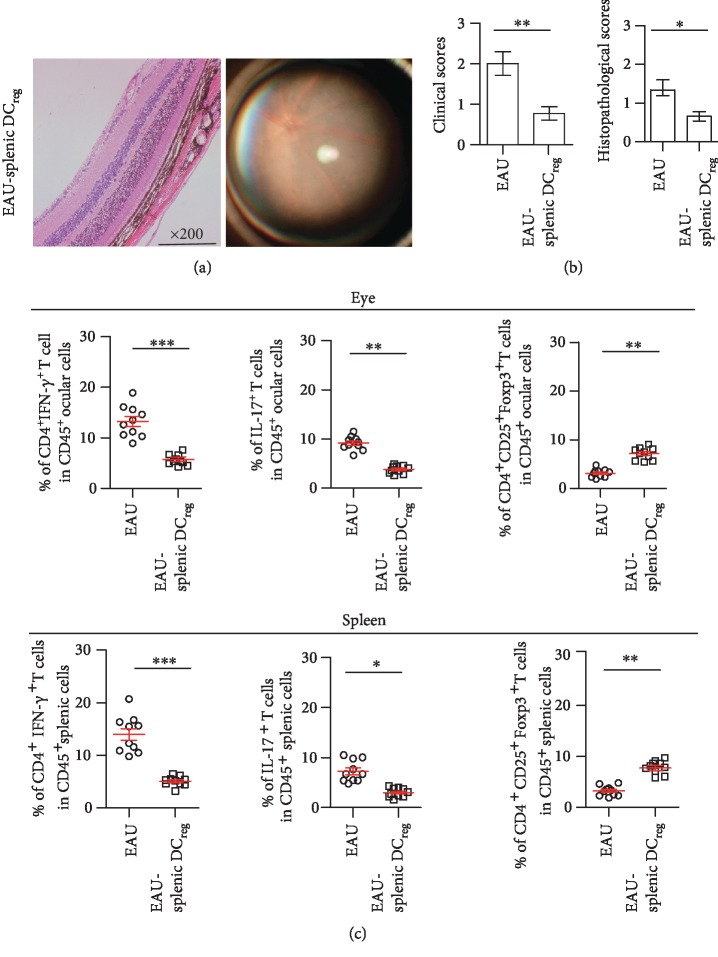
DC_reg_ isolated from the spleen of EAU decreased the symptom of EAU. DC_reg_ cells were isolated from the spleen of EAU and were transferred into the mice, which were immunized for 8 d. After 8 d of transferring, the symptom of the eyes was determined by ophthalmoscopy, and the eyes of those mice were obtained and were detected by H&E (a). Clinical scores and histopathological scores were analyzed (b). (c) The percentage of CD4^+^IFN-*γ*^+^ T cells, CD4^+^CD25^+^Foxp3^+^, or CD4^+^IL-17^+^ T cells in the eyes or spleen of splenic DC_reg_-transferred EAU mice compared with those without transfer. (*n* = 10/group, experiments were replicated three times, data were presented as mean ± standard error of the mean (SEM), two-tailed Student's *t*-test was conducted, and ^∗^*P* < 0.05, ^∗∗^*P* < 0.01, and ^∗∗∗^*P* < 0.001).

On the 28th day after immunization, the percentage of DC_reg_ also increased in the spleen of EAU (Supplementary [Supplementary-material supplementary-material-1]). The increased number of DC_reg_ may participate into the progression of EAU. DC_reg_ were isolated from the spleen of EAU mice, which were immunized for 28 days. These cells were transferred into EAU mice by intravenous injection. The severity of EAU was analyzed on the 8th day following DC_reg_ transferring. The retinal damage was not so serious in the eyes of splenic DC_reg_-transferred mice compared with those without transferring (Figure 4(a)). Both clinical and histopathological scores of the eyes of the splenic DC_reg_-transferred mice were decreased compared with those in EAU mice (Figure 4(b)). The percentage of CD4^+^IFN-*γ*^+^ T cells and CD4^+^IL-17^+^ T cells from the eyes or spleen of splenic DC_reg_-transferred mice decreased (Figure 4(c)). However, the percentage of CD4^+^CD25^+^Foxp3^+^ T cells in the eyes increased after splenic DC_reg_ transferring (Figure 4(c)).

The transferring splenic DC_reg_ was not present in the eyes tissues, although these cells were present in the spleen and lymph node (Supplementary [Supplementary-material supplementary-material-1]). The results indicated that DC_reg_ derived from the inflamed spleen could decrease the severity of EAU and could play a regulatory role in peripheral lymphoid organs. However, when DC_reg_ were isolated from the eyes of CD45.1-expressing mice and were transferred into EAU. These cells could reach to the inflamed eyes (Supplementary [Supplementary-material supplementary-material-1]). Above all, these data indicated that the function of DC_reg_ in EAU is organ specificity.

### 3.5. IFN-*γ* Correlated with the Differentiation of CD11c^low^I-a^low^CD11b^high^ DCs

**Figure 5:**
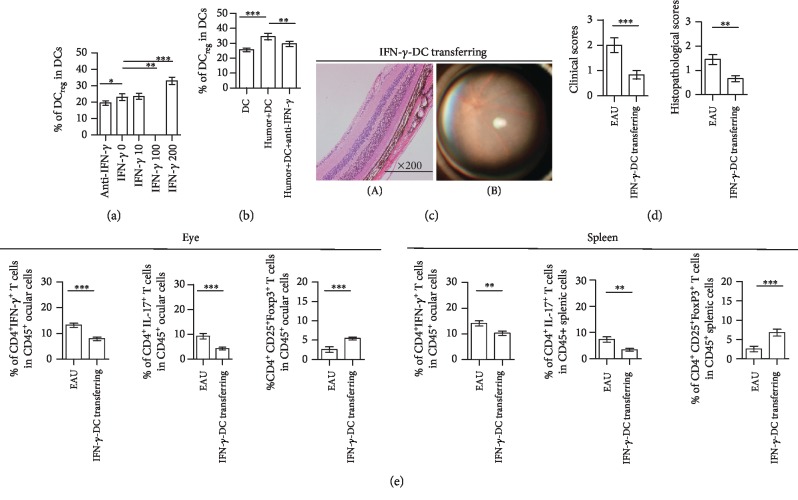
The effect of IFN-*γ* on DCs in vitro and IFN-*γ*-treated DCs on EAU. (a) With different concentrations of IFN-*γ* treatment or anti-IFN-*γ*-neutralizing antibodies (2 *μ*g/ml), the percentage of DC_reg_ in DCs isolated from the eyes of normal mice is shown. Mouse IFN-*γ* was used as 0 U/ml (IFN-*γ* 0), 10 U/ml (IFN-*γ* 10), 100 U/ml (IFN-*γ* 100), and 200 U/ml (IFN-*γ* 200) separately. The experiment was replicated three times, data were presented as mean ± standard error of the mean (SEM), ANOVA test, and ^∗^*P* < 0.05, ^∗∗^*P* < 0.01, and ^∗∗∗^*P* < 0.001. (b) The percentage of DC_reg_ in DCs, which with aqueous humor or aqueous humor added with anti-IFN-*γ*-neutralizing antibody (2 *μ*g/ml) compared with those without treatment. The experiment was replicated three times, data were presented as mean ± standard error of the mean (SEM), ANOVA test, and ^∗∗^*P* < 0.01. (c) Histopathological change of eyes (A) and clinical symptom (B) were assessed in IFN-*γ*-treated DC-transferred mice by H&E staining and funduscopy on the 16th d of postimmunization. H&E staining of the retina at 200x magnification. Scale bar = 100 *μ*m. (d) The histopathological and clinical scores were evaluated in IFN-*γ*-treated DC-transferred mice, compared with those of nontreated EAU mice. (e) The percentage of CD4^+^IFN-*γ*^+^ T cells, CD4^+^CD25^+^Foxp3^+^ T cells, or CD4^+^IL-17^+^ T cells in CD45^+^ lymphocytes of eyes and spleen of IFN-*γ*-treated DC-transferred mice compared with that of nontreated EAU mice. (d, e) *n* = 10/group, and experiments were replicated three times, data were presented as mean ± standard error of the mean (SEM), two-tailed Student's *t*-test, and ^∗^*P* < 0.05, ^∗∗^*P* < 0.01, and ^∗∗∗^*P* < 0.001.

To further analyze which factors influence the status of DCs in eye, we investigated the changed composition in aqueous humor in the process of EAU. In the inflamed aqueous humor, the concentration levels of IFN-*γ* increased during the recovery stage (Supplementary [Supplementary-material supplementary-material-1]). IFN-*γ* was reported to induce the production of the tolerogenic dendritic cells [46–48], and it was shown to exert a protective role in uveitis [49, 50]. Thus, the increased level of IFN-*γ* in the inflamed eyes might affect the status of DCs. The effect of IFN-*γ* on DCs was analyzed *in vitro*. The highest concentration of IFN-*γ* correlated with higher percentages of DC_reg_ (Figure 5(a)), whereas the highest concentration of IFN-*γ* promoted the expression of IDO by DC_reg_ (Supplementary [Supplementary-material supplementary-material-1]). The aqueous humors from EAU (21-28 days following immunization) were obtained and added to the culture of isolated ocular DCs, and the percentage of DC_reg_ was increased accordingly (Figure 5(b)). However, following treatment with the IFN-*γ* neutralizing antibody, the percentage of DC_reg_ was lower than those animals without treatment (Figure 5(b)). With IFN-*γ* neutralizing antibody treatment, the inflammatory symptoms in the eyes of the mice were aggravated (Supplementary [Supplementary-material supplementary-material-1]). The percentage of CD4^+^IL-17^+^ T cells increased, whereas the ratio of DC_reg_/DC_m_ and the percentage of CD4^+^CD25^+^Foxp3^+^ T cells decreased in the inflamed eyes and inflamed spleen. In contrast to these findings, the percentage of CD4^+^IFN-*γ*^+^ T cells was not changed significantly (Supplementary [Supplementary-material supplementary-material-1]). The data indicated that IFN-*γ* correlated with the differentiation of DC_reg_.

Furthermore, we pretreated isolated DCs for 72 h with IFN-*γ* (200 U/ml). These DCs (10^6^/ml) were transferred into EAU mice on day 8 following immunization. After transferring for 8 days, the severity of EAU was evaluated. The retinal damage was not found in the eyes of IFN-*γ*-treated DC_reg_-transferred mice (Figure 5(c)). Both clinical and histopathological scores of the eyes of IFN-*γ*-treated DC_reg_-transferred mice decreased compared with those of the EAU mice (Figure 5(d)).

After IFN-*γ*-treated DC_reg_ transfer, the number of lymphocyte subsets in mice was analyzed. The percentages of CD4^+^IFN-*γ*^+^ T cells and CD4^+^IL-17^+^ T cells from the eyes and spleen of DC_reg_-transferred mice were all decreased, compared with those from the mice without treatment (Figure 5(e)). However, the percentage of CD4^+^CD25^+^Foxp3^+^ T cells in the eyes was increased after IFN-*γ*-treated DC_reg_ transfer. Similar results were found in the spleen of IFN-*γ*-treated DC_reg_-transferred mice.

### 3.6. I-a^low^CD11b^high^ DCs Exist in the Eyes of Normal Mice and the Ocular Microenvironment Maintains the Tolerance State of DCs

**Figure 6:**
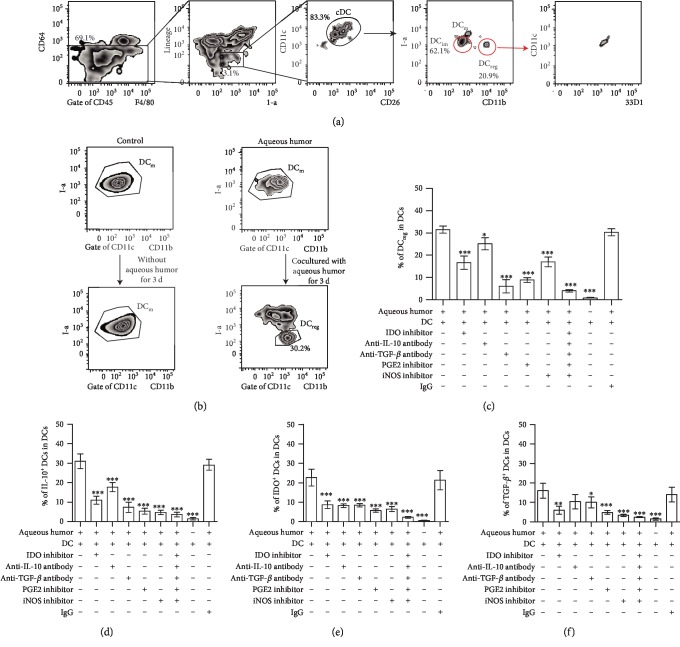
The status of DCs in the eyes of normal mice. (a) In addition to macrophages and monocyte-derived cells, CD3^+^ T cells, CD19^+^ B cells, and NK1.1^+^ natural killer (NK) cells (lineage mix) were also excluded. The representative I-a^low^CD11b^high^ DCs (DC_reg_) in ocular cells from normal eyes were detected by flow cytometry. (b) The representative diagram of I-a^low^CD11b^high^ regulatory DCs was in the culturation of DC_m_. I-a^high^CD11b^low^ DCs (DC_m_) were isolated and were stimulated by aqueous humor (100 *μ*l/ml, right) or not (control). After 3 days, DC_reg_ was detected in the culturation of DC_m_. (c) The percentage of DC_reg_ in the culturation of DC_m_, which is with aqueous humor added with anti-IL-10 antibody, anti-IDO antibody, anti-TGF-*β* antibody, anti-NO antibody, anti-PGE2, or the whole antibody, compared with those without treatment. DCs were isolated from the spleen of normal mice. (d–f) The percentage of IL-10^+^ DCs, IDO^+^ DCs, and TGF-*β*^+^ DCs in the culturation of DC_m_, which is with anti-IL-10 antibody, anti-TGF-*β* antibody, IDO inhibitor, NO inhibitor, PGE2 inhibitor, or the whole antibody or inhibitor stimulation, were added, compared with those without treatment. (d–f) Experiments were replicated three times, data were presented as mean ± standard error of the mean (SEM), and ^∗^*P* < 0.05, ^∗∗^*P* < 0.01, and ^∗∗∗^*P* < 0.001.

To further analyze whether DC_reg_ exist in the eyes of normal mice, CD45^+^ ocular cells from normal eyes were gated and analyzed. In these CD11c^+^CD26^+^DCs, 20.9% of DCs were DC_reg_. 62.1% of DCs were immature DCs (Figure 6(a)). Furthermore, depletion of ocular resident CD11c+DCs with DT in CD11c-DTR-GFP mice could promote the emergence of early inflammatory symptoms EAU (Supplementary [Supplementary-material supplementary-material-1]). However, DC_reg_ were isolated from the normal eyes of CD45.1-expressing mice and were transferred into EAU mice. The clinical and histopathological scores of the eyes of DC_reg_-transferred mice were decreased (Supplementary [Supplementary-material supplementary-material-1]). These data indicated that DC_reg_ existed in the normal eyes, and they might play a regulatory role in eye.

It was reported that aqueous humor is important for sustaining the tolerance of immune microenvironment [20–22, 51]. NO, IDO, prostaglandin E2 (PGE2), TGF-*β*, and IL-10 were reported to be present in aqueous humor [52–54], and the expression levels of all of these markers were associated with the status of DCs [4, 5, 8, 18, 55]. To analyze whether the composition of aqueous humor influence the status of ocular DCs, we obtained the aqueous humor from the normal eyes and detected the concentration levels of aforementioned soluble factors in the aqueous humor to determine whether these molecules could affect the DC status. The levels of the inflammatory markers in the aqueous humor of normal eyes were measured and showed as follows: NO (7.8 ± 3.4 *μ*M), IDO (114 ± 40.9 ng/ml), PGE2 (1.6 ± 0.9 pg/ml), TGF-*β* (3800 ± 130.9 pg/ml), and IL-10 (40 ± 13.6 pg/ml) in aqueous humor. Subsequently, we investigated the mechanisms underlying the differentiation of DC_reg_ induced by aqueous humor. Aqueous humor samples were obtained from the eyes of normal mice and were added to the culture medium of mature DCs (100 *μ*l/ml). 32.5 ± 3.5% of mature DCs differentiated into DC_reg_, indicating that aqueous humors could affect the differentiation of DCs (Figure 6(b)).

To investigate which factors in aqueous humors affect DC differentiation, anti-IL-10, TGF-*β* neutralizing antibody, and PGE2, NO, and IDO inhibitor were used to block IL-10, TGF-*β*, PGE2, NO, and IDO expressions alone or in combination. The expression level assessment of these markers was performed in the DC coculture with aqueous humors stimulation. After incubation for 3 days, the percentage of DC_reg_ in mature DCs was detected. The percentage of DC_reg_ in the culture of mature DCs was decreased (Figure 6(c)). Moreover, in the presence of neutralizing antibody or inhibitors, the expression levels of IL-10, TGF-*β*, and IDO in DC_reg_ decreased (Figures 6(d)–6(f)), indicating that aqueous humors affected the status of DCs.

## 4. Discussion

The eye is considered as an organ that is immunologically privileged and possesses a phenotype with lacking lymphocytes [21, 56], but CD11c^+^DCs were found to exist in the eyes and might play important roles in tissue homeostasis and the immune response to foreign antigens [11–13, 56, 57]. However, the roles and status of DCs in the eyes are still not unclear. Herein, we demonstrated that the majority of ocular DCs were CD11c^+^CD26^+^DCs. I-a^low^CD11b^high^ DCs (DC_reg_) are regulatory subsets in the eyes of normal mice. These cells can express 33D1 and low levels of CD80, CD86, and CD54. Moreover, these cells can secret IL-10, TGF-*β*, and IDO, which might induce the ocular immune tolerance.

Uveitis is a serious inflammatory disease that can result in visual disability and blindness [24, 58]. In the inflammatory eye, the number of regulatory DCs was increased and achieved a peak during the recovery stage. These increased regulatory DCs may migrate to draining lymph nodes and the spleen to inhibit T-cell activation or induce CD4^+^CD25^+^Foxp3^+^ T cells [11, 56]. Our data demonstrated that regulatory DC might exert its inhibitory function with tissue specificity. Adoptively transferring splenic DC_reg_ into EAU mice could inhibit the inflammation of EAU by inducing CD4^+^CD25^+^Foxp3^+^ T cells. But these splenic DC_reg_ could not arrive to the inflamed eyes. Splenic DC_reg_ might play a regulatory role in peripheral lymphoid organs by inducing CD4^+^CD25^+^Foxp3^+^ T cells. Additionally, ocular DC_reg_ also regulated the immune response to influence the local and systemic immune status by secreting IL-10, TGF-*β*, and IDO.

The tissue microenvironment and the immunosuppressive cytokines can regulate and determine the functions of DCs *in vivo* [8, 10, 16, 59]. The aqueous humors, which contain a certain concentration of proteins, provide important substances to maintain the microenvironment of the eye and are secreted from the ciliary epithelium [20–22]. Specific levels of IL-10, TGF-*β*, NO, IDO, and PGE2 were found in the aqueous humor to sustain the state of regulatory DCs and drive the differentiation of mature DCs to regulatory DCs. These data suggested that the aqueous humor could influence the differentiation of DCs. IL-10 and TGF-*β* can be further elevated and maintained at high levels during the recovery period of EAU (Supplementary [Supplementary-material supplementary-material-1]). These cytokines might further induce the tolerance of T cells and DCs to promote the recovery of EAU.

Additionally, the increasing levels of IFN-*γ* in the eyes may further promote the differentiation of DC_reg_. The role of IFN-*γ* in autoimmunity is still controversial. IFN-*γ* was reported to have a protective role in EAU [50, 60]. Another study demonstrated that IFN-*γ* may promote the inflammation of EAU [61]. The role of IFN-*γ* may be associated with its concentration or the effective time on DCs, since high concentration and/or long treatment effect of IFN-*γ* on DCs could induce DC tolerance by promoting IDO expression [46]. In the inflamed eyes of EAU mice, the levels of IFN-*γ* revealed a sustained increase [62]. The increasing levels of IFN-*γ* positively correlated with the expression levels of IDO in DCs. IDO could further inhibit T-cell proliferation and suppress tissue injury [46–48, 63]. Therefore, the sustained increase of IFN-*γ* may be considered one of the main regulatory factors for the induction of DC_reg_ in EAU. Recurrent uveitis may be associated with the defect in the regulation of IFN-*γ* and/or the expression of IDO.

The mechanisms of action of DC_reg_ vary among the different tissue [8, 10, 16, 59]. DC_reg_-derived PGE2 was responsible for the regulatory function in the liver and pulmonary tissues [10, 16]. However, DC_reg_-derived NO was responsible for the regulatory function in the spleen [5]. Our data showed that DC_reg_ in the eyes could secret TGF-*β*, IL-10, and IDO, which may induce ocular immune tolerance. The increased number of DC_reg_ in the inflamed eyes may cause their migration into the lymph node or spleen to suppress T-cell activation by secreting inhibitory molecules or regulating cell-cell contact.

A limited number of therapy regimens have been discovered for the treatment of uveitis [64–66]. Numerous studies have focused on the development of customized and targeted immunotherapies, including cell-based therapies [64–66]. In the present study, we showed that DCs pulsed with IRBP_1-20_ and PTX and stimulated by IFN-*γ* could alleviate the severity of retinal damage in EAU and could decrease the number of infiltrated CD3^+^ T cells, NK cells, and DCs in the eyes and the inflamed spleen. The present study demonstrated that IFN-*γ*-treated DCs played a suppressive role in the inflammatory response of T cells. IFN-*γ*-treated DCs promoted the proliferation of Treg and inhibited the development of EAU by stimulating IDO expression and IFN-*γ*-treated DC cell-Treg cell interaction. Therefore, the transfer of IFN-*γ*-treated DCs is effective for the treatment of EAU. It might be helpful for us to seek a potential therapeutic strategy for autoimmune uveitis.

## 5. Conclusion

The current study concludes that DC_reg_ exists in normal and inflamed eyes and promotes CD4^+^CD25^+^Foxp3^+^ T cells by secreting IDO, IL-10, and TGF-*β*. The status of DC_reg_ is mainly regulated by aqueous humor, which is rich in TGF-*β*, NO, and PGE2. The high level of IFN-*γ* in the aqueous humor of the recovery stage of eyes promotes the number of regulatory DCs, which could alleviate the symptom of EAU. Our results suggest that DC_reg_ play a protective role in EAU, and these DC_reg_ induced by IFN-*γ* might be used as a strategy to develop therapy for EAU management.

## Data Availability

The data used to support the findings of this study are available from the corresponding author upon request.

## Supplementary Materials

Supplementary Materials Supplementary Figure S1: change of dynamic percentage of DC_reg_ and DC_m_ in CD45^+^ cells in the spleens of EAU. *n* = 15/group, the experiment was replicated three times, data were presented as mean ± standard error of the mean (SEM), Kruskal-Wallis test, and ^∗^*P* < 0.05, ^∗∗^*P* < 0.01, and ^∗∗∗^*P* < 0.001. Supplementary Figure S2: transferring DC_reg_ from inflamed spleen of CD45.1-expressing mice in the EAU; these cells appeared in the lymph node or spleen of a CD45.2-expressing EAU mice model, but not in the eyes. Supplementary Figure S3: the ocular DC_reg_ were adoptive transferred into EAU to arrive at the inflamed eyes. DC_reg_ from the eyes of CD45.1-positive mice were adoptive transferred into CD45.2-expressing EAU mice. After 12 days, the ocular cells were analyzed by flow cytometry. The representative pictures are shown. Supplementary Figure S4: the concentrations of IFN-*γ* in the aqueous humor of EAU mice after 28-day immunization, compared with those without immunization. *n* = 5/group, experiments were replicated three times, and data were presented as mean ± standard error of the mean (SEM). Two-tailed Student's *t*-test and ^∗∗∗^*P* < 0.001. Supplementary Figure S5: with different concentrations of IFN-*γ* treatment or anti-IFN-*γ*-neutralizing antibody treatment (2 *μ*g/ml), the percentage of IDO+DCs in the isolated ocular DCs. Mouse rIFN-*γ* was used as 0 U/ml (IFN-*γ* 0), 10 U/ml (IFN-*γ* 10), 100 U/ml (IFN-*γ* 100), and 200 U/ml (IFN-*γ* 200) separately. *n* = 15/group, the experiment was replicated three times, data were presented as mean ± standard error of the mean (SEM), ANOVA test, and ^∗∗∗^*P* < 0.001. Supplementary Figure S6: IFN-*γ*-neutralizing antibody treatment aggravated the symptoms of EAU. (a) Clinical scores and histopathological scores of the eyes from IFN-*γ*-neutralizing antibody-treated mice were analyzed and compared with those without treatment. The percentage of CD4^+^IFN-*γ*^+^ T cells, CD4^+^IL-17^+^ T cells, CD4^+^CD25^+^Foxp3^+^, and DCs in the eyes (b) or spleen (c) of IFN-*γ*-neutralizing antibody-treated mice, compared with those without treatment (*n* = 5/group, experiments were replicated three times, data were presented as mean ± standard error of the mean (SEM), two-tailed Student's *t*-test was conducted, and ^∗∗∗^*P* < 0.001, ^∗∗^*P* < 0.01, and ^∗^*P* < 0.05). Supplementary Figure S7: depletion of CD11c^+^ cells with diphtheria toxin (DT), the symptom of EAU decreased. (a) With DT depletion, the CD11c^+^ cells were analyzed in the eyes of CD11c-DTR-GFP, compared with that without depletion. (b) CD11c^+^ cells were depleted by DT, and then IPBR and PTX were injected to construct the EAU model. The histopathological scores of inflamed eyes were analyzed every four days. Data were presented as mean ± standard error of the mean (SEM), Kruskal-Wallis test, and ^∗^*P* < 0.05. Supplementary Figure S8: the role of the ocular DC_reg_ from normal eyes. The histopathological scores and clinical scores of EAU with or without transferring DC_reg_ from normal eyes of CD45.1-expressing mice. Data were presented as mean ± standard error of the mean (SEM), ANOVA test, and ^∗^*P* < 0.05 and ^∗∗^*P* < 0.01. Supplementary Figure 9: the dynamic concentration changes of TGF-*β* and IL-10 in the serum of EAU were determined by ELISA. *n* = 5/group, experiments were replicated three times, and data were presented as mean ± standard error of the mean (SEM). (Supplementary Materials).
