# Corrigendum to “I-a^low^CD11b^high^ DC Regulates the Immune Response in the Eyes of Experimental Autoimmune Uveitis”

**DOI:** 10.1155/2020/2637019

**Published:** 2020-11-28

**Authors:** Yu Zhao, Jingwen Wang, Zhang Min, Li Peng, Shuhong Qi, Wei Lin

**Affiliations:** ^1^Institute of Basic Medicine, Shandong Provincial Hospital Affiliated to Shandong First Medical University, Institute of Basic Medicine, Shandong First Medical University & Shandong Academy of Medical Schools, Jinan City 250062, China; ^2^The Third Affiliated Hospital of Shandong First Medical University, Affiliated Hospital of Shandong Academy of Medical Schools, 38# Wuyingshan Road, Jinan City 250031, China; ^3^Departments of Medicine, Tibet Nationalities University, Xian City 712082, China; ^4^Britton Chance Center for Biomedical Photonics, Wuhan National Laboratory for Optoelectronics-Huazhong University of Science and Technology, Wuhan, Hubei 430074, China; ^5^MoE Key Laboratory for Biomedical Photonics, Collaborative Innovation Center for Biomedical Engineering, School of Engineering Sciences, Huazhong University of Science and Technology, Wuhan, Hubei 430074, China

In the article titled “I-a^low^CD11b^high^ DC regulates the immune response in the eyes of experimental autoimmune uveitis” [1], affiliation 1 was incorrect and the correct affiliation is shown above. The authors also requested to correct a typographical error in the Methods and Materials section as follows:

“EAU in C57BL/6 mice was inducted by the 350 *μ*g of human interphotoreceptor retinoid-binding protein peptide (IRBP)1-20 (China Peptides Co.,Ltd., Shanghai, China) emulsified in complete Freund's adjuvant with mycobacterium (CFA, Sigma-Aldrich, St. Louis, MO, USA). A total of 500 ng of Pertussis toxin (PTX, Enzo Life Sciences, Farmingdale, YN, USA) was intraperitoneally injected at the footpad, neck, two sides, and tail at six points for every mouse as previously described [35]”

should be corrected as follows:

“EAU in C57BL/6 mice was inducted by the 350 *μ*g of human interphotoreceptor retinoid-binding protein peptide (IRBP)_1-20_ (China Peptides Co., Ltd., Shanghai, China) emulsified in complete Freund's adjuvant with mycobacterium (CFA, Sigma-Aldrich, St. Louis, MO, USA) and was immunized at six different locations (footpads, neck, two sides, and tail). Concurrently, a total of 500 ng of pertussis toxin (PTX, Enzo Life Sciences, Farmingdale, NY, USA) was injected intraperitoneally for every mouse as previously described [35].”
