# Supplementary material for: I-alowCD11bhigh DC Regulates the Immune Response in the Eyes of Experimental Autoimmune Uveitis
Source: Mediators Inflamm. 2020 Mar 10;2020:6947482. doi: 10.1155/2020/6947482 (PMC7085850; doi:10.1155/2020/6947482)
Supplement: Supplementary Materials — Supplementary Figure S1: change of dynamic percentage of DCreg and DCm in CD45+ cells in the spleens of EAU. n = 15/group, the experiment was replicated three times, data were presented as mean ± standard error of the mean (SEM), Kruskal-Wallis test, and ∗P < 0.05, ∗∗P < 0.01, and ∗∗∗P < 0.001. Supplementary Figure S2: transferring DCreg from inflamed spleen of CD45.1-expressing mice in the EAU; these cells appeared in the lymph node or spleen of a CD45.2-expressing EAU mice model, but not in the eyes. Supplementary Figure S3: the ocular DCreg were adoptive transferred into EAU to arrive at the inflamed eyes. DCreg from the eyes of CD45.1-positive mice were adoptive transferred into CD45.2-expressing EAU mice. After 12 days, the ocular cells were analyzed by flow cytometry. The representative pictures are shown. Supplementary Figure S4: the concentrations of IFN-γ in the aqueous humor of EAU mice after 28-day immunization, compared with those without immunization. n = 5/group, experiments were replicated three times, and data were presented as mean ± standard error of the mean (SEM). Two-tailed Student's t-test and ∗∗∗P < 0.001. Supplementary Figure S5: with different concentrations of IFN-γ treatment or anti-IFN-γ-neutralizing antibody treatment (2 μg/ml), the percentage of IDO+DCs in the isolated ocular DCs. Mouse rIFN-γ was used as 0 U/ml (IFN-γ 0), 10 U/ml (IFN-γ 10), 100 U/ml (IFN-γ 100), and 200 U/ml (IFN-γ 200) separately. n = 15/group, the experiment was replicated three times, data were presented as mean ± standard error of the mean (SEM), ANOVA test, and ∗∗∗P < 0.001. Supplementary Figure S6: IFN-γ-neutralizing antibody treatment aggravated the symptoms of EAU. (a) Clinical scores and histopathological scores of the eyes from IFN-γ-neutralizing antibody-treated mice were analyzed and compared with those without treatment. The percentage of CD4+IFN-γ+ T cells, CD4+IL-17+ T cells, CD4+CD25+Foxp3+, and DCs in the eyes (b) or spleen (c) of IFN-γ-neutralizing a [file 6947482.f1.pdf]

I-a^{low}CD11b^{high} DC regulates the immune response in the eyes of experimental autoimmune uveitis

Yu Zhao[1*], Jingwen Wang[2*], Zhang Min[3], Li Peng[1], Shuhong Qi[4], Wei Lin[1#]

[1] Institute of Basic Medicine, Shandong First Medical University & Shandong Academy of Medical Schools, Jinan, 250032, China.

[2] Affiliated Hospital of Shandong First Medical University & Shandong Academy of Medical Schools, 38# Wuyingshan Road, Jinan City, 250031, China.

[3] Departments of Medicine, Tibet Nationalities University, Xian City, 712082, China

[4] Britton Chance Center for Biomedical Photonics, Wuhan National Laboratory for Optoelectronics-Huazhong University of Science and Technology, Wuhan, Hubei, 430074, China; MoE Key Laboratory for Biomedical Photonics, Collaborative Innovation Center for Biomedical Engineering, School of Engineering Sciences, Huazhong University of Science and Technology, Wuhan, Hubei, 430074, China.

*co-first author

Correspondence should be addressed to Wei Lin; linw1978@163.com or weilin11@fudan.edu.cn

Supplementary Figure legends

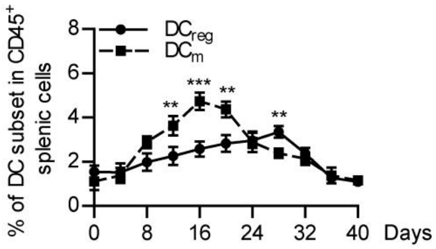

Supplementary Figure S1. Change of dynamic percentage of DC_{reg} and DC_m in $CD45^+$ cells in the spleens of EAU. n = 15/group, the experiment was replicated three times, data were presented as mean ± standard error of mean (SEM), Kruskal-Wallis test, *$P < 0.05$, ** $P < 0.01$, ***$P < 0.001$.

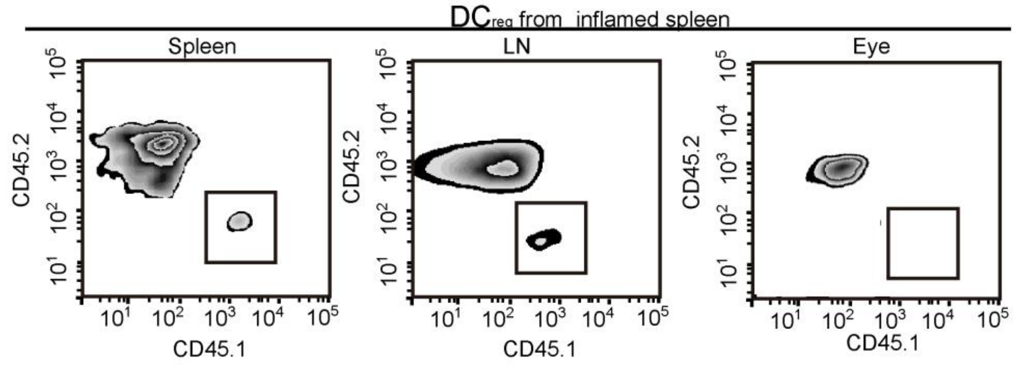

Supplementary Figure S2. Transferring DC_{reg} from inflamed spleen of CD45.1-expressing mice in the EAU, these cells appeared in lymph node (LN) or spleen of CD45.2- expressing EAU mice model, but not in the eyes.

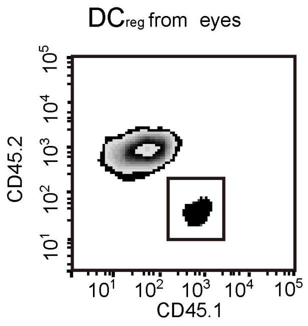

Supplementary Figure S3. The ocular DC_{reg} were adoptive transferred into EAU to arrive the inflamed eyes. DC_{reg} from the eyes of CD45.1 positive mice were adoptive transferred into CD45.2-expressing EAU mice. After 12 days, the ocular cells were analyzed by flow cytometry. The representative pictures were shown.

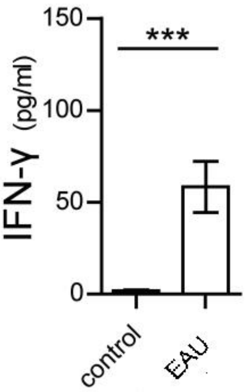

Supplementary Figure S4. The concentrations of IFN-γ in the aqueous humor of EAU mice after 28 day immunization, compared with these without immunized. n = 5/group, experiments were replicated three times, and data were presented as mean ± standard error of mean (SEM). Two-tailed Student's t-test, ***$P < 0.001$.

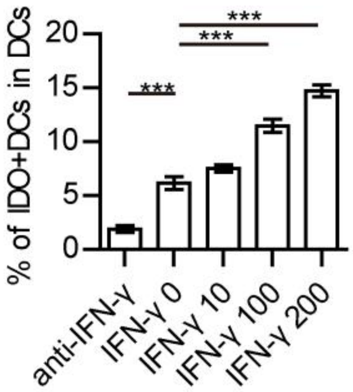

Supplementary Figure S5. With different concentrations of IFN-γ treatment,or anti-IFN-γ neutralizing antibodies treatment (2μg/ml), the percentage of IDO^{+}DCs in the isolated ocular DCs. Mouse rIFN-γ was used as 0U/ml (IFN-γ 0), 10U/ml (IFN-γ 10), 100U/ml (IFN-γ 100) and 200U/ml (IFN-γ 200) separately. n = 15/group, the experiment was replicated three times, data were presented as mean ± standard error of mean (SEM), ANOVA test, $***P < 0.001$

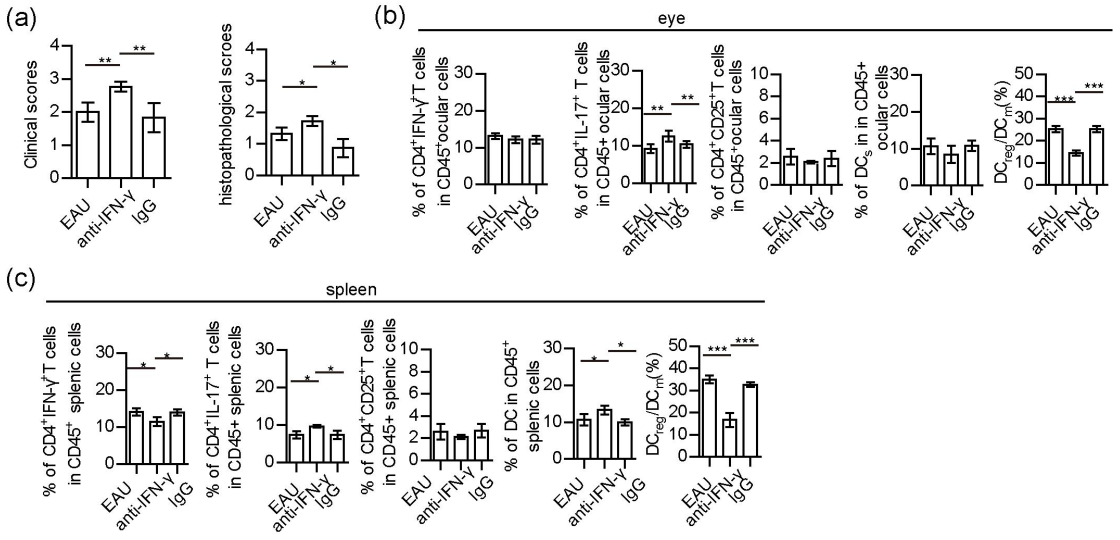

Supplementary Figure S6. IFN-γ neutralizing antibody treatment aggravated the symptoms of EAU. (a). Clinical scores and histopathological scores of the eyes from IFN-γ neutralizing antibody treated mice were analyzed, and compared with these without treatment. The percentage of CD4$^+$IFN-γ$^+$T cells, CD4$^+$IL-17$^+$T cells, CD4$^+$CD25$^+$Foxp3$^+$ and DCs in the eyes (b) or spleen (c) of IFN-γ neutralizing antibody treated mice, compared with these without treatment (n = 5/group, experiments were replicated three times, data were presented as mean ± standard error of mean (SEM), two-tailed Student's t-test was conducted, and ***$P < 0.001$, **$P < 0.01$, *$P < 0.05$).

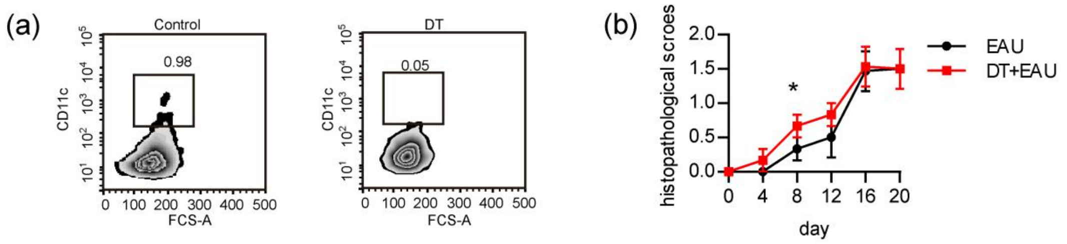

Supplementary Figure S7. Depletion of CD11c$^+$ cells with diphtheria toxin (DT), the symptom of EAU decreased. (a) With DT depletion, the CD11c+ cells were analyzed in the eyes of CD11c-DTR-GFP, compared with that without depletion. (b) CD11c+ cells were depleted by DT, and then IPBR and PTX were injected to construct the EAU model. The histopathological scores of inflamed eyes were analyzed every four days. Data were presented as mean ± standard error of mean (SEM), Kruskal-Wallis test, *$P < 0.05$.

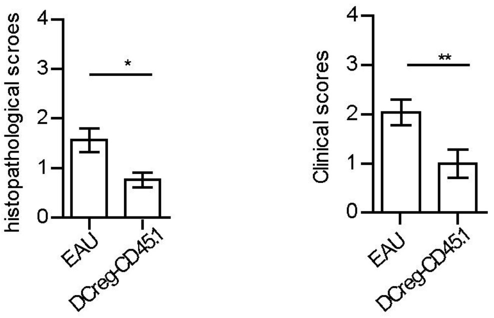

Supplementary Figure S8. The role of ocular DC_{reg} from normal eyes. The histopathological scores and clinical scores of EAU with or without transferring DC_{reg} from normal eyes of CD45.1-expressing mice. Data were presented as mean ± standard error of mean (SEM), ANOVA test, **P* < 0.05, ***P* < 0.01.

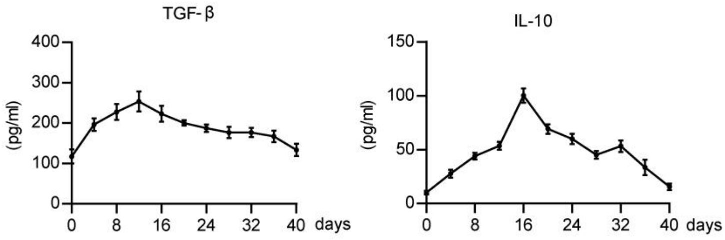

Supplementary Figure 9. The dynamic concentrations changes of TGF-β and IL-10 in the serum of EAU were determined by ELISA. n = 5/group, experiments were replicated three times, and data were presented as mean ± standard error of mean (SEM).

.
